# The comparison of short-term outcomes between robotic and laparoscopic radical distal gastrectomy

**DOI:** 10.1007/s00423-023-02866-9

**Published:** 2023-03-29

**Authors:** Yang Song, Qianshi Zhang, Zhen Feng, Bo Wang, Shuangyi Ren

**Affiliations:** Department of General Surgery, The Second Hospital of Dalian Medical University, Dalian, 116023 China

**Keywords:** Gastric cancer, Minimally invasive surgery, Robotic, Laparoscopic, Learning curve

## Abstract

**Purpose:**

The study’s objectives were to compare the short-term outcomes of robotic radical distal gastrectomy (RDG) with laparoscopic radical distal gastrectomy (LDG) for patients with gastric cancer and investigate the learning curve of RDG.

**Methods:**

The cumulative sum (CUSUM) method was used to retrospectively analyze consecutive gastric cancer patients undergoing RDG between January 2019 and October 2021. The duration of surgery, clinical-pathological characteristics, and short-term outcomes were evaluated according to the two phases of the learning curve (learning period versus mastery period). We also compared the clinical-pathological characteristics and short-term outcomes between cases in the mastery period and LDG.

**Results:**

Data from 290 patients were included in this analysis, 135 RDG and 155 LDG cases. The learning period was 20 cases. There were no significant differences in clinical-pathological characteristics between the learning period and mastery period. Compared with the learning period, the mastery period had a significant reduction in total operation time, docking time, pure operation time, and estimated blood loss, and a significant increase in hospital costs (*P*=0.000, 0.000, 0.000, 0.003, and 0.026, respectively). Compared with LDG, robotic cases in mastery period had a longer operative time, shorter first postoperative flatus time, and more hospital costs (*P*=0.000, 0.005, and 0.000, respectively).

**Conclusions:**

RGD may fasten to recover gastrointestinal function faster after the operation, can be mastered easily after a reasonable number of cases, and was associated with safe and satisfactory short-term outcomes before and after the learning curve.

## Introduction

Gastric cancer is the third leading causes of cancer death worldwide [1]. One of the surgical approaches for gastric cancer is minimally invasive surgery, such as laparoscopic and robotic surgery. Laparoscopic distal gastrectomy shows benefits in terms of low complication rate, fast recovery, and less pain [2], and did not result in inferior 3 years of disease-free survival [3]. The robotic surgical system is a new minimally invasive surgical platform. Of them, the da Vinci surgical system is the most widely employed in clinical practice, provides 3D imaging with high definition, articulated movement, and elimination of physiologic tremor [4]. Some studies have shown that robotic radical distal gastrectomy can achieve better short-term outcomes. Such as obtaining more lymph nodes [5], significantly reducing blood loss [6], postoperative complications and additional injuries [7, 8], and shortening postoperative hospital stay [9].

Meanwhile, other studies have shown that there are no significant differences from those of laparoscopic surgery [10–12]. As opposed, there are also disadvantageous factors such as prolonged operative time [13] and increased hospitalization costs [14]. The different short-term results of robotic radical distal gastrectomy may root in that the robotic procedure needs time to conquer the learning curve. However, there are few literatures that have reported the short-term data on robotic surgery after overcoming the learning curve and then compared it with laparoscopic procedures.

The purpose of this study was to analyze the learning curve of robotic radical distal gastrectomy (RDG) and then compare the short-term outcomes with laparoscopic radical distal gastrectomy(LDG) in the same period to assess the benefit of RDG.

## Materials and methods

### Patients

**Fig. 1:**
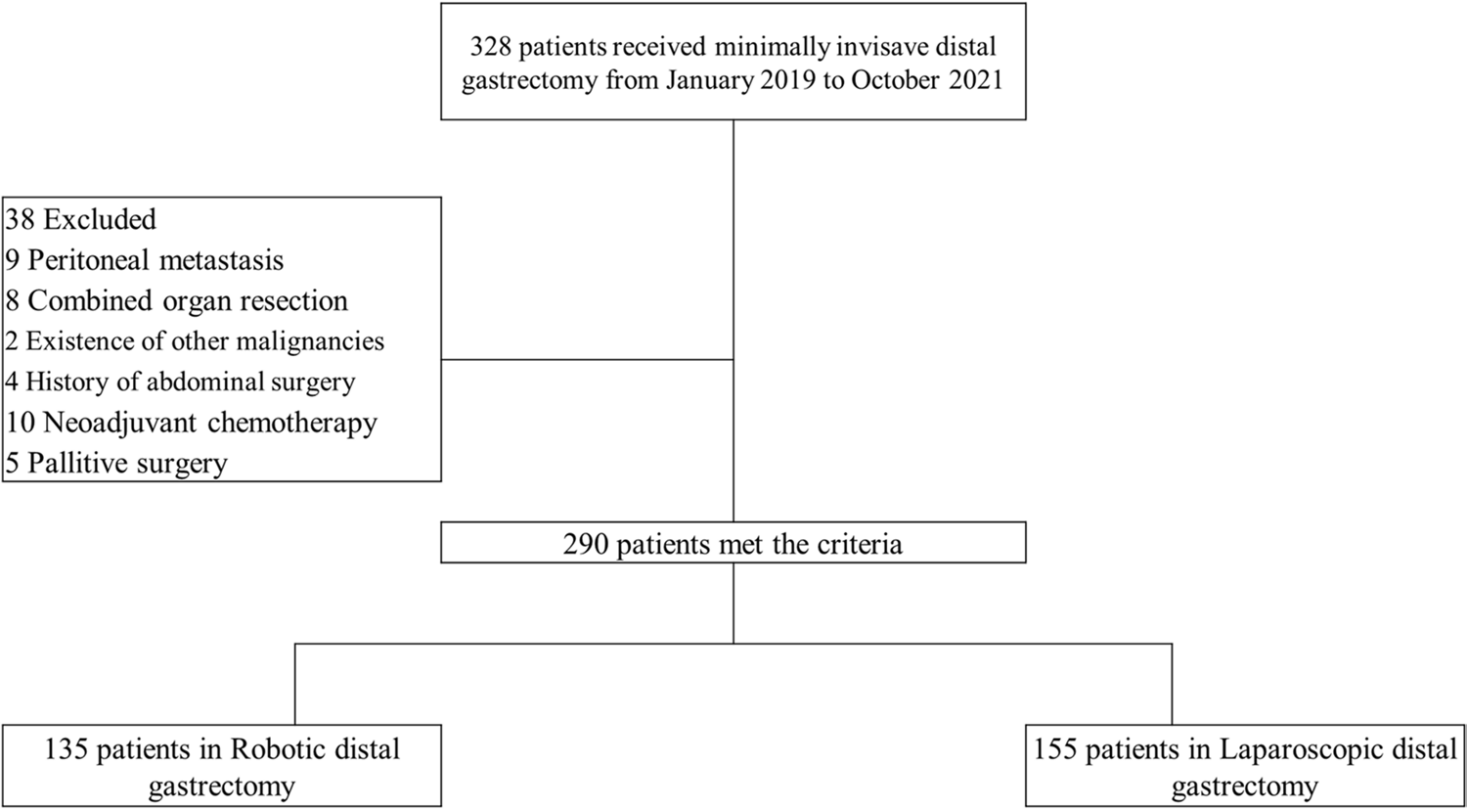
The flow diagram of the study patient selection process

This was a retrospective study of data obtained from January 2019 to October 2021 at The Second Affiliated Hospital of Dalian Medical University. A total of 328 patients who received minimally invasive distal gastrectomy were recruited in this study. At the same period, we also conducted 32 cases of robotic total gastrectomy (RTG) and 36 cases of laparoscopic total gastrectomy (LTG) respectively. Thirty-eight of these patients were excluded, including 9 patients with peritoneal metastasis, 8 patients combined organ resection, 2 patients with existence of other malignancies, 4 patients with a history of abdominal surgery, 10 patients with Neoadjuvant chemotherapy, and 5 patients got palliative surgery. A total of 135 patients were divided into the robotic distal gastrectomy group, and the other 155 patients were into the laparoscopic gastrectomy group. Figure 1 showed the flow diagram of the study patient selection process. All the cases were operated by a single surgeon (Shuangyi Ren), who had performed laparoscopic gastrectomy in more than 1500 cases before performing totally robotic distal gastrectomy. Cancer staging was performed based on the findings of contrast-enhanced computed tomography. The patients with clinical T≤4a without Bulky lymph node were determined to be resectable. Before surgery, all patients were well informed about the comparison between the robotic and laparoscopic gastrectomy, and patients who chose robotic surgery would receive robotic radical distal gastrectomy, whereas the remaining patients would undergo laparoscopic radical distal gastrectomy. All the patients personally signed the consent. Medical records were extracted from the prospectively maintained database at the Department of Gastrointestinal Surgery. This study was approved by the Ethics Committee of The Second Affiliated Hospital of Dalian Medical University.

### Inclusion and exclusion criteria

All the patients who underwent RDG were included. Patients with (1) distant metastasis; (2) existence of other malignancies; (3) history of abdominal surgery; (4) history of gastric cancer treatment by endoscopic resection, chemotherapy and/or radiotherapy; (5) severe cardiopulmonary, hepatic, and renal insufficiency; (6) emergency surgery; (7) combined organ resection; and (8) palliative surgery were excluded.

### Surgical procedure

In our center, after exploring the abdominal cavity, all the patients who received radical distal gastrectomy would undergo standard D2 lymphatic dissection according to the Japanese gastric cancer treatment guideline 2014 [15] either by robotic or laparoscopic approach. However, for patients with clinical T stage less than 3, we preferred to reserve the greater omentum. The digestive tract reconstruction was chosen Billroth I, Billroth II, Billroth II+Braun or Roux-en-Y, taking into account the tumor position, the remnant stomach, and anastomotic tension. All the lymphatic tissue was divided by the surgeon according to the Japanese classification of gastric carcinoma: 3rd English edition, as follows: No.6; No.4d; No.4sb; No.5/12a; No.7/8a/9/11p; No.1/3, and then sent to the pathology department for lymph node retrieved and diagnosed. Postoperative pathology was staged according to the 7th edition of the American Cancer Consortium (AJCC) gastric cancer staging system [16]. Postoperative complications were graded according to the Clavien-Dindo classification [17]. Due to the docking time during RDG procedure, we analyzed the surgical time using total operation time and pure operation time. The total operation time was defined as the time from the start of the abdominal incision through the completion of closure of the wounds, which including pure operation time and docking time. The pure operation time was defined as the time from the start of the surgeon use the console to move any articulating surgical instruments through the completion of closure of the wounds.

### Perioperative care

Diet started with water on the first postoperative day, then processed to a soft diet according to the patient’s reaction. Patients could be discharged from the hospital if they were in good condition on the sixth or seventh postoperative day. Diet schedule, flatus, and other conditions were recorded daily until discharge.

### CUSUM and statistical analysis

All patients were listed consecutively according to the date of surgery and calculated using the following formula $\textrm{CUSUM}=\sum \limits_{\textrm{i}=1}^{\textrm{n}}\left(\textrm{Xi}-\mu \right)$, Xi means the operation time of the da Vinci surgical system for each case, μ means the average operation time of all robotic radical distal gastrectomy patients, and *n* means the patient serial number. The scatter plot of the learning curve was shown with the number of surgical cases as the horizontal coordinate and the CUSUM value as the vertical coordinate. After plotting the CUSUM learning curve, the point where a decline occurred was the starting point of the data below the mean for that case, and the horizontal coordinate corresponding to that point was the number of surgical cases necessary to pass the learning period. SPSS (SPSS Inc., version 26.0, Chicago, IL, USA) was used for statistical analysis. Continuous variables were expressed as mean ± standard deviation (mean ± SD) and the *t* test or Mann-Whitney test was chosen for comparison between groups. Metric variables were expressed as number (n), the χ2 test or Fisher’s exact probability test was selected for the count data. Statistical significance was set at *p* < 0.05.

### Ethics statement

All the patients personally signed the consent. Medical records were extracted from the prospectively maintained database at the Department of Gastrointestinal Surgery. This study was approved by the Ethics Committee of The Second Affiliated Hospital of Dalian Medical University.

## Results

All the patients (RDG 135 vs. LDG 155) received the surgical procedure, neither RDG patients nor LDG patients conversed to open surgery and no patient died during the hospital stay.

### CUSUM analysis of the RDG learning curve

**Fig. 2:**
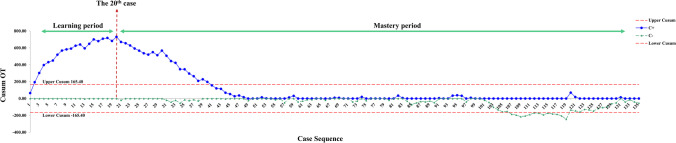
Learning curve for robotic radical distal gastrectomy

CUSUM analysis was introduced to analyze the operation time, and it showed a gradual increase till the 20th case, then it has gone down until the 49th case (Fig. 1). So, the learning curve was considered as 20 cases in the present study (Fig. 2). The RDG cases were divided into two groups: the learning period (1st–20th) and the mastery period (21st–135th).

### Comparison of patients’ characteristics and short-term outcomes before and after learning curve

All data according to the learning curve are shown in Table 1. Although there was more lymph node metastasis in the learning period, there were no significant differences in clinical-pathological characteristics between the learning and the mastery period. All data for operative and postoperative are shown in Table 2. Compared with the learning period, even though the estimated blood loss was significantly decreased in the mastery period (107.25±86.11 versus 59.96±36.61 ml, *p*=0.003). Total operation time, docking time, and pure operation time were significantly shorter in the mastery period (257.40±49.05 versus 173.14±32.74 min, *p*<0.001; 40.00±6.88 versus 22.91±3.38 min, *p*<0.001; 217.40±44.76 versus 150.23±31.65 min, *p*<0.001; respectively). The hospitalization cost in the mastery period was significantly higher than in the learning period (93286±9139 versus 99313±18070 CNY, p= 0.026).

**Table 1:** Comparisons of characteristics between the learning and mastery period

Variables	The learning period *n*=20 (case 1 to 20)	The mastery period *n*=115 (case 21 to 135)	*p*
Age (years)	65.10 ± 6.35	63.80 ± 9.70	0.564
Gender			0.465
Male	16 (80%)	83 (72.2%)	
Female	4 (20%)	32 (27.8%)	
BMI (kg/m^2^)	23.90 ± 2.85	24.10 ± 3.15	0.792
Tumor size (cm)	3.78 ± 1.46	3.53 ± 2.22	0.215
Histologic grade†			0.214
G1	0	12 (10.4%)	
G2	6 (30.0%)	45 (39.2%)	
G3	14 (70.0%)	58 (50.4%)	
Vessel invasion	9 (45.0%)	53 (46.1%)	0.928
Nerve invasion	9 (45.0%)	32 (27.8%)	0.123
pT stage*			0.404
T1/Tis	6 (30.0%)	53 (46.0%)	
T2	4 (20.0%)	14 (12.2%)	
T3	3 (15.0%)	21 (18.3%)	
T4a	7 (35.0%)	27 (23.5%)	
PN stage*			0.065
N0	7 (35.0%)	67 (58.3%)	
N1	4 (20.0%)	12 (10.4%)	
N2	6 (30.0%)	12 (10.4%)	
N3a	2 (10.0%)	11 (9.6%)	
N3b	1 (5.0%)	13 (11.3%)	

The data were expressed as mean ± standard deviation or number (%)

*BMI,* body mass index

†Histologic grade, G1, well differentiated; G2, moderately differentiated; G3, poorly differentiated

*The stages were classified by the 7^th^ UICC/AJCC staging system

**Table 2:** Surgical outcomes and postoperative course comparisons between the learning and mastery period

Variables	The learning period *n*=20 (case 1 to case 20)	The mastery period *n*=115 (case 21 to case 135)	*p*
Total operation time (min)	257.40 ± 49.05	173.14 ± 32.74	<0.001
Docking time (min)	40.00 ± 6.88	22.91 ± 3.38	<0.001
Pure operation time (min)	217.40 ± 44.76	150.23 ± 31.65	<0.001
Estimated blood loss (ml)	107.25 ± 86.11	59.96 ± 36.61	0.003
Postoperative complications	3 (15%)	13 (11.30%)	0.707
Pneumonia	2	5	
Delayed gastric emptying	1	2	
Bowel obstruction	0	1	
Wound infection	0	2	
Anastomotic leakage	0	1	
Intra-abdominal bleeding	0	1	
Intra-abdominal abscess	0	1	
Major complications*	2 (10%)	7 (6.10%)	0.622
Pneumonia	1	3	
Delayed gastric emptying	1	2	
Bowel obstruction	0	1	
Intra-abdominal bleeding	0	1	
Postoperative stay (days)	8.25 ± 3.46	7.56 ± 3.44	0.092
First flatus (days)	2.55 ± 0.61	2.43 ± 0.61	0.422
First defecation (days)	3.25 ± 1.07	3.37 ± 0.81	0.270
First liquid diet (days)	2.90 ± 2.36	2.04 ± 0.95	0.053
Harversted lymph nodes	32.10 ± 15.25	32.70 ± 12.06	0.843
Positive lymph nodes	5.40 ± 12.19	3.49 ± 6.54	0.130
Total hospitalization cost (CNY)	93286 ± 9139	99313 ± 18070	0.026
Anastomosis method			0.150
Billroth I	9 (45.00%)	29 (25.20%)	
Billroth II	7 (35.00%)	41 (35.70%)	
Billroth II+Braun	4 (20.00%)	29 (25.20%)	
Roux-en-Y	0	16 (13.90%)	

The data were expressed as mean ± standard deviation or number (%)

*Major complications were defined according to the Clavian-Dindo classification greater than or equal to IIIa

The following major complications were observed in the learning period: pneumonia with pleural fluid (*n*=1), which was managed using radiological intervention (Grade IIIa); delayed gastric emptying (*n*=1), which was managed using endoscopic intervention (Grade IIIa). The following major complications were observed in the mastery period: pneumonia with pleural fluid (*n*=3), which were managed using radiological intervention (Grade IIIa); delayed gastric emptying (*n*=2) and bowel obstruction (*n*=1), which were managed using endoscopic intervention (Grade IIIa); intra-abdominal bleeding (*n*=1) which required reoperation (Grade IIIb). Although there were no statistically significant differences in postoperative complications or the proportion of complications of Clavien-Dindo grade III and above, it showed a decreasing trend in the mastery period. Finally, more patients received Roux-en-Y anastomosis in the mastery period (0 versus 13.9%).

### Comparison of patients’ characteristics and short-term outcomes between mastery period and LDG

Compared with LDG, there were no significant differences in clinical-pathological characteristics in the mastery period of RDG (Table 3).

**Table 3:** Comparisons of clinical-pathological characteristics between the mastery period of RDG and LDG

Variables	The mastery period of RDG *n*=115 (case 21 to 135)	LDG	*p*
Age (years)	63.80 ± 9.70	65.55 ± 10.10	0.191
Gender			0.062
Male	83 (72.2%)	95 (61.3%)	
Female	32 (27.8%)	60 (38.7%)	
BMI (kg/m^2^)	24.10 ± 3.15	24.08 ± 5.03	0.389
Tumor size (cm)	3.54 ± 2.22	3.80 ± 1.90	0.057
Histologic grade^†^			0.571
G1	12 (10.4%)	13 (8.4%)	
G2	45 (39.2%)	54 (34.8%)	
G3	58 (50.4%)	88 (56.8%)	
Vessel invasion	53 (46.1%)	72 (46.5%)	0.953
Nerve invasion	32 (27.8%)	53 (34.2%)	0.265
PT stage*			0.491
T1/Tis	53 (46.0%)	59 (38.0%)	
T2	14 (12.2%)	27 (17.4%)	
T3	21 (18.3%)	32 (20.6%)	
T4a	27 (23.5%)	37 (24.0%)	
PN stage*			0.268
N0	67 (58.3%)	75 (48.3%)	
N1	12 (10.4%)	15 (9.7%)	
N2	12 (10.4%)	24 (15.5%)	
N3a	11 (9.6%)	26 (16.8%)	
N3b	13 (11.3%)	15 (9.7%)	

The data were expressed as mean ± standard deviation or number (%)

*BMI* body mass index

†Histologic grade, G1, well differentiated; G2, moderately differentiated; G3, poorly differentiated

* The stages were classified by the 7^th^ UICC/AJCC staging system

Although compared with LDG, the total operation time was significantly longer in the mastery period of RDG (173.14±32.74 versus 155.14±41.89 min, *p*<0.001), the pure operation time was comparable with LDG (150.23±31.65 versus 155.14±41.89 min, *p*=0.703). The first flatus time was significantly shorter in the mastery period of RDG than in LDG (2.43±0.61 versus 2.70±0.79d, *p*= 0.005). However, the hospitalization cost in the mastery period of RDG was significantly higher than LDG (99314±18070 versus 82143±21713 CNY, *p*<0.001) (Table 4). The following major complications were observed in the LDG: pneumonia with pleural fluid (*n*=2), anastomotic leakage (*n*=2) and duodenal stump leakage (*n*=1) which were managed using radiological intervention (Grade IIIa); delayed gastric emptying (*n*=1) and bowel obstruction (*n*=1), which were managed using endoscopic intervention (Grade IIIa); multiple organ dysfunction (*n*=2) required ICU management (Grade IVb). Compared with the mastery period of RDG, there was no statistically significant differences in postoperative complications or the proportion of complications of Clavien-Dindo grade III and above. There were no statistically significant differences in other characteristics between the two groups.

**Table 4:** Surgical outcomes and postoperative course comparisons between the mastery period of RDG and LDG

Variables	The mastery period of RDG *n*=115 (case 21 to 135)	LDG	*p*
Total operation time (min)	173.14 ± 32.74	155.14 ± 41.89	<0.001
Pure operation time (min)	150.23 ± 31.65	155.14 ± 41.89	0.703
Estimated blood loss (ml)	59.96 ± 36.61	86.42 ± 198.22	0.596
Postoperative complications	13 (11.30%)	29 (18.70%)	0.097
Pneumonia	5	8	
Delayed gastric emptying	2	5	
Bowel obstruction	1	1	
Wound infection	2	3	
Anastomotic leakage	1	5	
Duodenal stump leakage	0	1	
Intra-abdominal bleeding	1	2	
Intra-abdominal abscess	1	0	
Multiple organ dysfunction	0	2	
Myelosuppression	0	2	
Major complications*	7 (6.10%)	9 (5.80%)	0.923
Pneumonia	3	2	
Delayed gastric emptying	2	1	
Bowel obstruction	1	1	
Intra-abdominal bleeding	1	0	
Anastomotic leakage	0	2	
Duodenal stump leakage	0	1	
Multiple organ dysfunction	0	2	
Postoperative stay (days)	7.56 ± 3.44	8.91 ± 6.62	0.467
First flatus (days)	2.43 ± 0.61	2.70 ± 0.79	0.005
First defecation (days)	3.37 ± 0.81	3.56 ± 1.22	0.324
First liquid diet (days)	2.04 ± 0.95	2.94 ± 3.36	0.175
Harvested lymph nodes	32.70 ± 12.06	35.94 ± 13.65	0.058
Positive lymph nodes	3.49 ± 6.54	4.91 ± 8.16	0.069
Total hospitalization cost (CNY)	99314 ± 18070	82143 ± 21713	<0.001
Anastomosis method			0.190
Billroth I	16 (13.90%)	10 (6.50%)	
Billroth II	29 (25.20%)	49 (31.60%)	
Billroth II+Braun	41 (35.70%)	56 (36.10%)	
Roux-en-Y	29 (25.20%)	40 (25.80%)	

The data were expressed as mean ± standard deviation or number (%)

*Major complications were defined according to the Clavian-Dindo classification greater than or equal to IIIa

## Discussion

In 1994, Kitano [18] first reported laparoscopic-assisted radical gastric cancer surgery, and laparoscopic surgery has recently gained popularity because of its advantages such as minimally invasive, less surgery-related complications, faster postoperative recovery, and shorter hospital stay. The results of existing studies show that for patients with early gastric cancer, the short- and long-term outcomes of robotic surgery system are comparable to those of laparoscopic surgery.

However, the mastery of robotic radical distal gastrectomy requires a number of surgical cases to be accumulated. In this study, based on the operation time, we used the CUSUM method to analyze the learning curve of robotic radical distal gastrectomy, and the results showed that the learning curve was 20 cases, which was similar to the learning curve of robotic surgery systems reported in existing related studies [19].

When comparing the total operation time, the docking time, the pure operation time, and the estimated blood loss between the patients in the learning period and the mastery period, there were statistically significant differences. While other surgical variables between the two groups, there were no statistically significant differences. The results were attributed to that after the learning period, skilled surgeons can take full advantage of the robotic systems, such as filtering the physiologic tremor, accurate and stable arms, and reducing ineffective movement to reduce the operation time and blood loss. Furthermore, the inevitable docking time in robotic procedures can be dramatically reduced along with familiarity with the robotic systems. Meanwhile, the hospitalization cost in the mastery period was more than that of the learning period, which may due to the lower proportion of Roux-en-Y anastomosis in the learning period. As the process of Roux-en-Y anastomosis costs more surgical materials, such as linear stapler cartridges and absorbable sutures, which ultimately lead to an increase in total hospitalization cost.

The highlights of the study, we introduced the CUSUM method to analyze the learning curve of robotic radical distal gastrectomy, then analyzed the comparison between the mastery period data with LDG, which maximally reduced the deviation stemming from the unfamiliarity of the robotic system, making the data comparable to the laparoscopic group. The results showed that the total operation time was significantly longer in the mastery period compared to LDG, which is consistent with the results of most current studies [13, 20, 21]. After culling the docking time in this study, the pure operation time of the robotic procedure was not longer than laparoscopic significantly. So we analyzed the reason for consuming more surgical time in robotic procedures mainly resulting from the docking time. Some previous studies reported that the 3D high-definition image and the flexible instruments of the robotic system allow the surgeon to dissect the lymphatic tissue along the intricate anatomical structures more clearly and easily, especial in difficult areas, such as in the inferior pyloric area and the superior pancreatic border [22], easy to reduce intraoperative bleeding [20, 23]. However, there was no significant difference in the harvested lymph nodes and estimated intraoperative bleeding between the mastery period and LDG, which is not consistent with the results of previous studies [24, 25]. The reason may be that the surgical procedures of robotic are the same as those of laparoscopic, and the surgeons in this study have extensive experience in laparoscopic surgery, which helped the surgeons moved the laparoscopic instruments in abdominal precisely and freely, that makes less ineffective operation time and less accessory injury to reduce the bleeding. At the same time, cooperation with an experienced assistant can also get better surgery field manifestation. Those may offset some of the advantages of robotic technology. We also find that the major complications do not differ between the mastery period of RDG and LDG, which is consistent with the results of previous study [26]. What’s more, the harvested lymph nodes whether in the robotic group or in the laparoscopic group are comparable with the previous randomized clinical trials (references CLASS01). In addition, the first postoperative flatus time was shorter in the mastery period compared with the LDG, suggesting that the robotic procedure is more conducive to postoperative gastrointestinal tract function recovery, which is consistent with some existing studies [12]. About the higher hospitalization cost in the mastery period, which results from the use of the robotic instruments and the additional sterile protective shield. With the high cost, advantages to use robotic system for the expert surgeon of LDG are still remarkable. Firstly, the robotic system can release the limitation about the number of surgeons. During robotic surgery, one surgeon and an assistant can meet the demand. However, more surgeons are necessary when performing the laparoscopic surgery. Secondly, the robotic system is more ergonomics, it gives comfort to surgeons performing complex procedure, which may reduce the damage of surgeons’ upper limb joints and may prolong the surgeons’ service life. Furthermore, comfortable surgical environment may result in high-quality surgical outcomes.

In this study, there were some limitations. Firstly, although the lymphadenectomy procedures were performed by a single surgeon, the reconstruction was not performed using robotic instruments but laparoscopic linear staplers. Thus, it depends on the assistant skill. Second, all the cases were finished by a single surgeon, so there may contain some deviations in identifying the benefits of robotic radical distal gastrectomy compared with laparoscopically. Finally, this was a single institution, retrospective cohort study, although the data were gained prospectively. The benefits of robotic radical distal gastrectomy remain to be approved by further randomized clinical trials.

## Conclusions

Robotic radical distal gastrectomy can be mastered easily after a reasonable number of cases and was associated with safe and satisfactory short-term outcomes before and after the learning curve. At the same time, it is conducive to patients’ postoperative gastrointestinal tract function recovery after surgery.
